# Supplementary material for: Evidence for a trap-and-flip mechanism in a proton-dependent lipid transporter
Source: Nat Commun. 2022 Feb 23;13:1022. doi: 10.1038/s41467-022-28361-1 (PMC8866510; doi:10.1038/s41467-022-28361-1)
Supplement: Supplementary file 1 — Supplementary Information [file 41467_2022_28361_MOESM1_ESM.pdf]

Supplementary materials for

Evidence for a trap-and-flip mechanism in a proton-dependent lipid transporter

Elisabeth Lambert, Ahmad Reza Mehdipour, Alexander Schmidt, Gerhard Hummer, Camilo Perez*

*Correspondence should be addressed to C.P. (email: camilo.perez@unibas.ch)

This PDF file includes:

Figs. S1 to S8
Tables S1 to S2

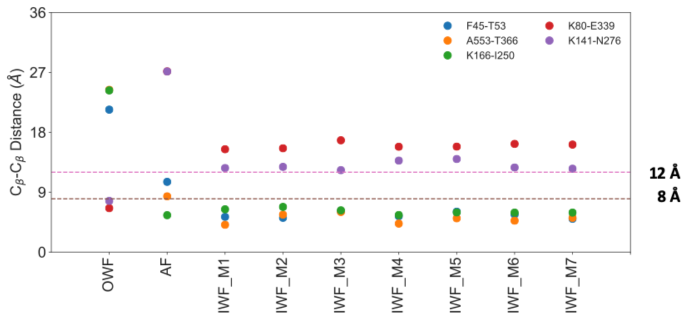

Residues	OWF	AF	IWF_M1	IWF_M2	IWF_M3	IWF_M4	IWF_M5	IWF_M6	IWF_M7
F45-T253	21.4	10.5	5.3	5.2	6.2	5.4	6.1	5.7	5.0
A53-T366	24.4	8.4	4.1	5.7	6.1	4.3	5.1	4.8	5.2
K166-I250	24.3	5.5	6.4	6.8	6.3	5.7	6.0	6.0	6.0
K80-E339	6.7	27.1	15.5	15.6	16.8	15.8	15.8	16.2	16.1
K141-N276	7.6	27.1	12.7	12.8	12.4	13.8	14.0	12.8	12.5

Supplementary figure 1. Distances between residues selected for cross-linking in alternating conformations of LtaA. Cβ-Cβ distances between residues selected for cysteine cross-linking in the outward-facing structure (OWF), in the best inward-facing models (INW_M), and in the AlphaFold (AF) inward-facing model. Distance thresholds considered for selection of residues pairs are shown.

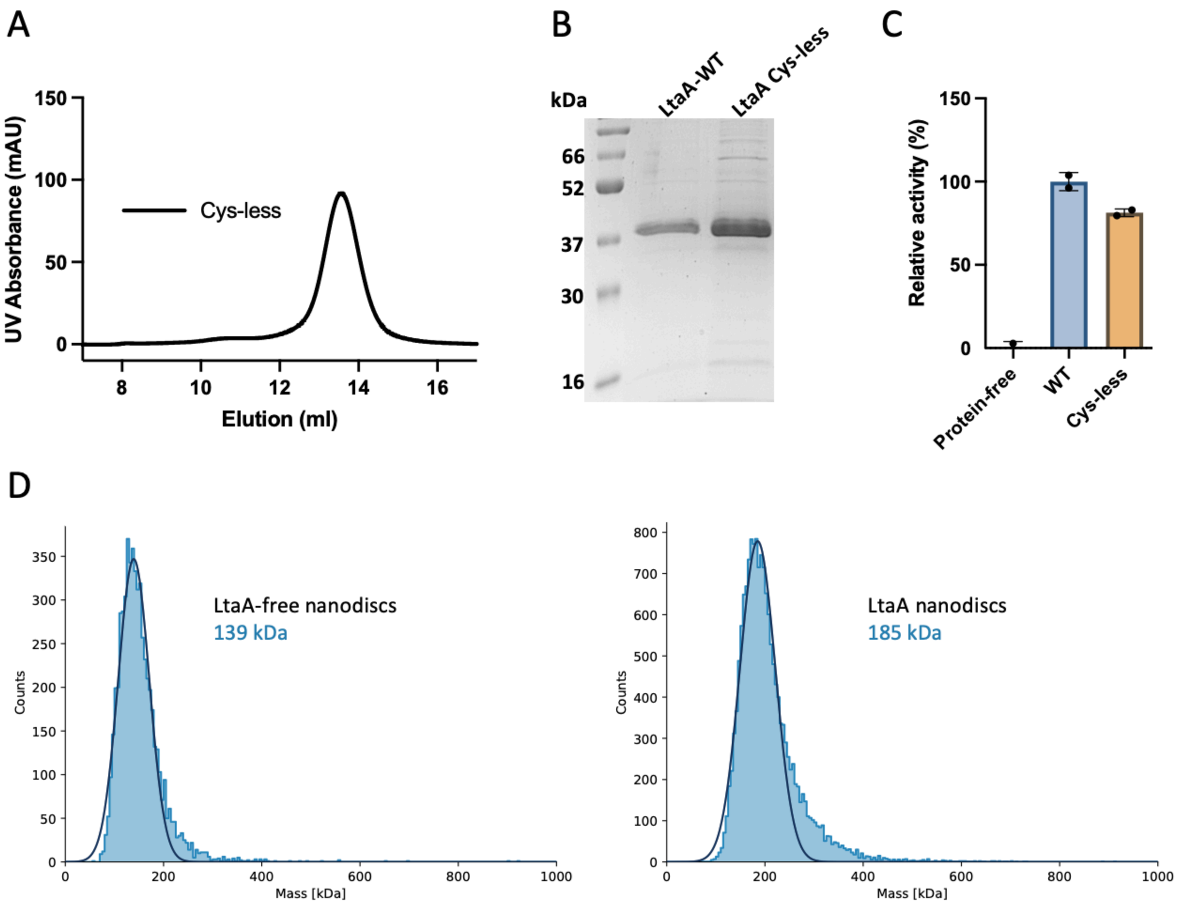

Supplementary figure 2. Analysis of LtaA wild-type and cysteine-less variant. A. Size Exclusion Chromatography profile of cysteine-less LtaA in a Superdex 200 Increase 10/300 GL column. **B**. SDS-PAGE of purified LtaA-WT and LtaA Cys-less. **C**. Relative flipping activity of LtaA Cys-less and LtaA-WT. Error bars show +/- standard deviation (s.d.) of technical replicates, n=3. **D**. Mass photometry analysis of nanodiscs without LtaA (*left*) and LtaA-reconstituted nanodiscs (*right*). The approximate molecular mass averages from Gaussian distributions are shown. The resulting apparent molecular mass difference (LtaA mass) is 46 kDa.

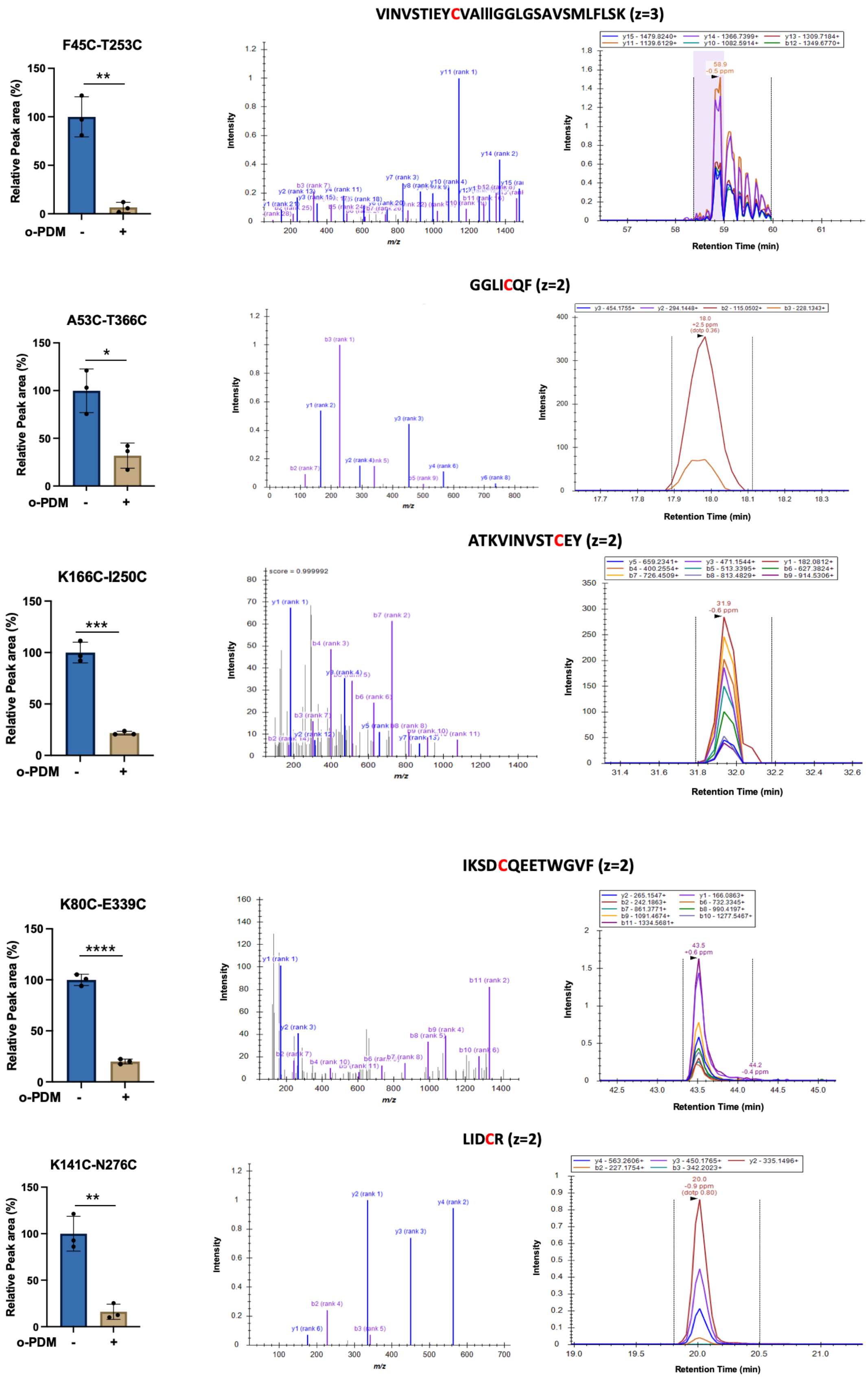

Supplementary figure 3. LC-MS analysis of cysteine cross-linking showing that LtaA adopts inward- and outward-facing states. Selected pairs of residues for cross-linking are shown in Fig. 2. *Left*: Relative abundance of cysteine containing peptides in absence (-) or presence (+)

of N,N'-(o-phenylene)-dimaleimide (o-PDM). Error bars indicate +/- s.d. (n=3, biological replicates). *: P≤0.05, **: P≤0.01, ***: P≤0.001, ****: P≤0.0001. Asterisks mark the result from unpaired t-test. *Center*: Assigned Collision-Induced Dissociation (CID) spectra obtained from cysteine containing peptides in absence of o-PDM. The position of the alkylated cysteine is indicated in red and the charge state (z) of the identified peptide ion is provided. *Right*: Elution profiles of the peptide fragments (transitions) identified used for peptide quantification by targeted LC-MS analysis. Source data are provided as a Source Data file.

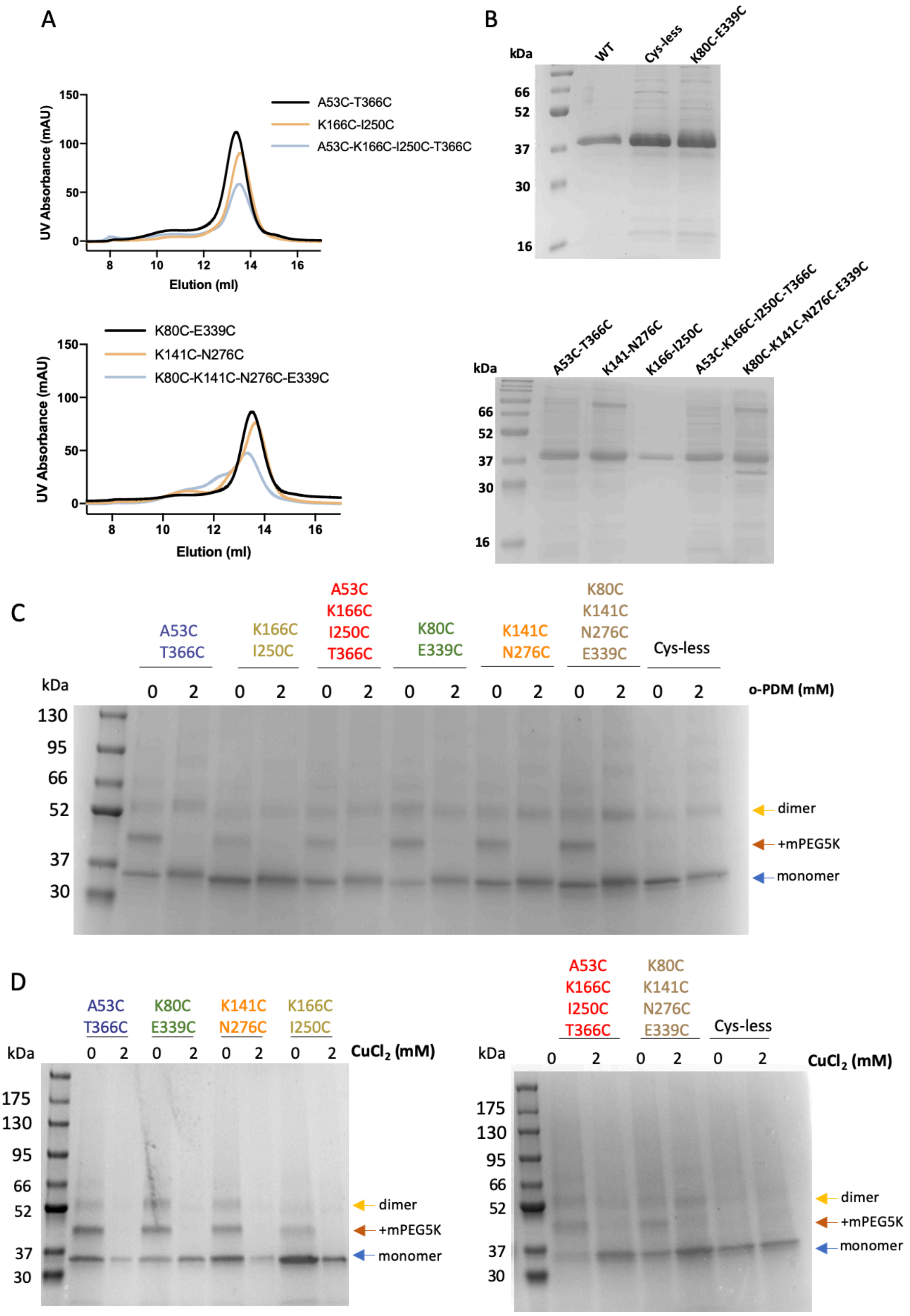

Supplementary figure 4. Purifications and cross-linking in-gel analysis of LtaA variants. A. Size Exclusion Chromatography profile of LtaA variants in a Superdex 200 Increase 10/300 GL column. **B**. SDS-PAGE of purified LtaA WT and variants. **C** and **D**. SDS-PAGE showing band shifts of samples treated with mPEG5K after cross-link with o-PDM or $CuCl_2$, respectively. SDS-PAGE experiments were independently repeated at least three times with similar results. Source Data are provided as a Source Data file.

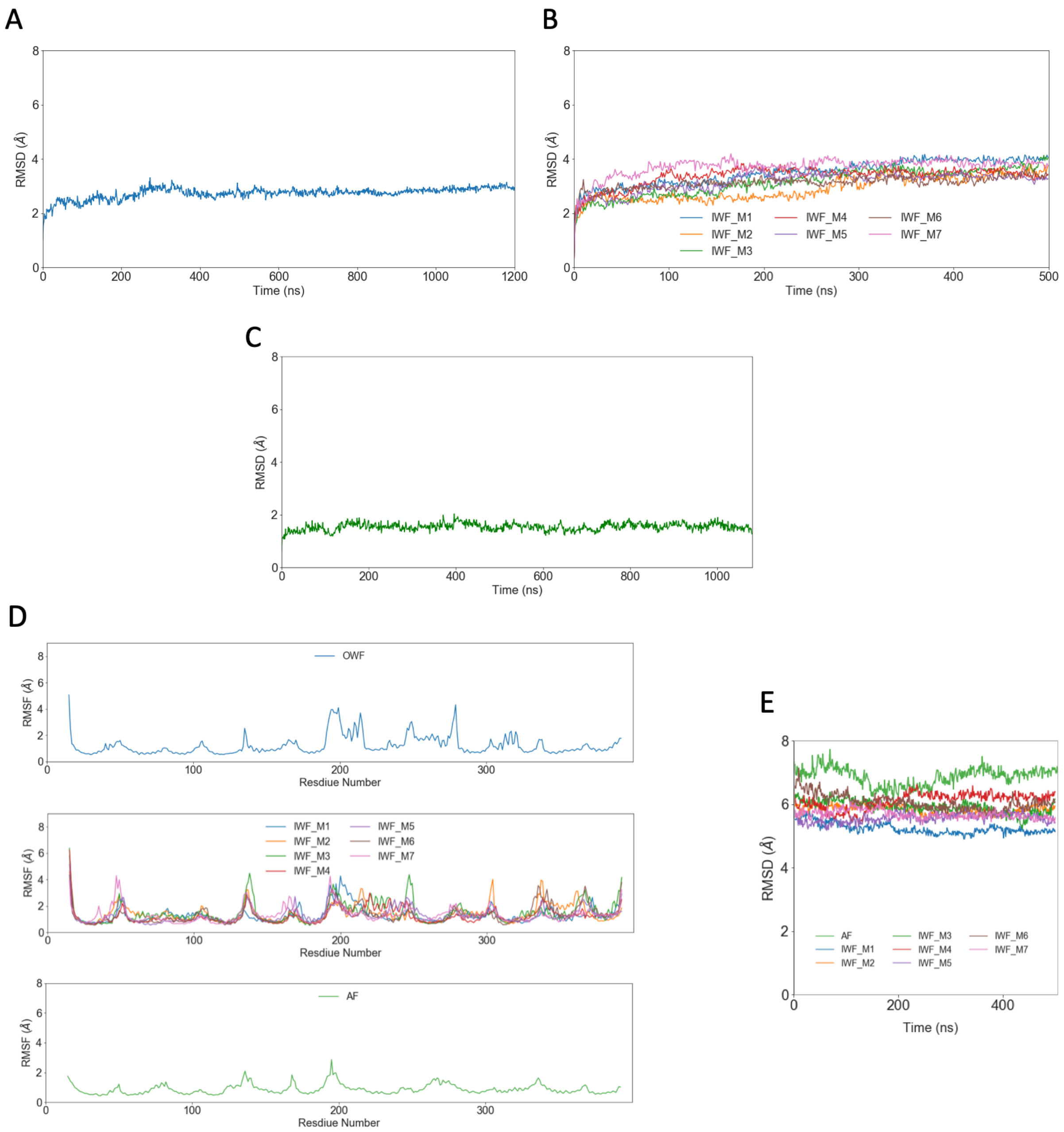

Supplementary figure 5. Stability analysis from molecular dynamics simulations of LtaA in a membrane. **A-C**. Root-mean-square-deviation (RMSD) plots of Cα atoms relative to their starting points during MD simulations in a membrane composed of POPG (65%), diacylglycerol (20 %), cardiolipin (10 %), and gentiobiosyl-diacylglycerol (5 %). RMSD plots of Cα atoms for outward-facing LtaA (**A**), inward-facing LtaA models obtained from the 'repeat-swap' approach (IWF_M) (**B**), and the inward-facing LtaA model generated by AlphaFold (AF) (**C**) are shown. **D**. Root-mean-square-fluctuation (RMSF) plot of Cα atoms for outward-facing (OWF) LtaA, 'repeat-swap' inward-facing LtaA models, and the inward-facing LtaA model generated by AlphaFold (AF), calculated for each MD simulation based on the average structure. **E**. RMSD plot of Cα atoms of 'repeat-swap' inward-facing LtaA models and the AF inward-facing LtaA model during MD simulations relative to the outward-facing structure (PBD ID 6S7V).

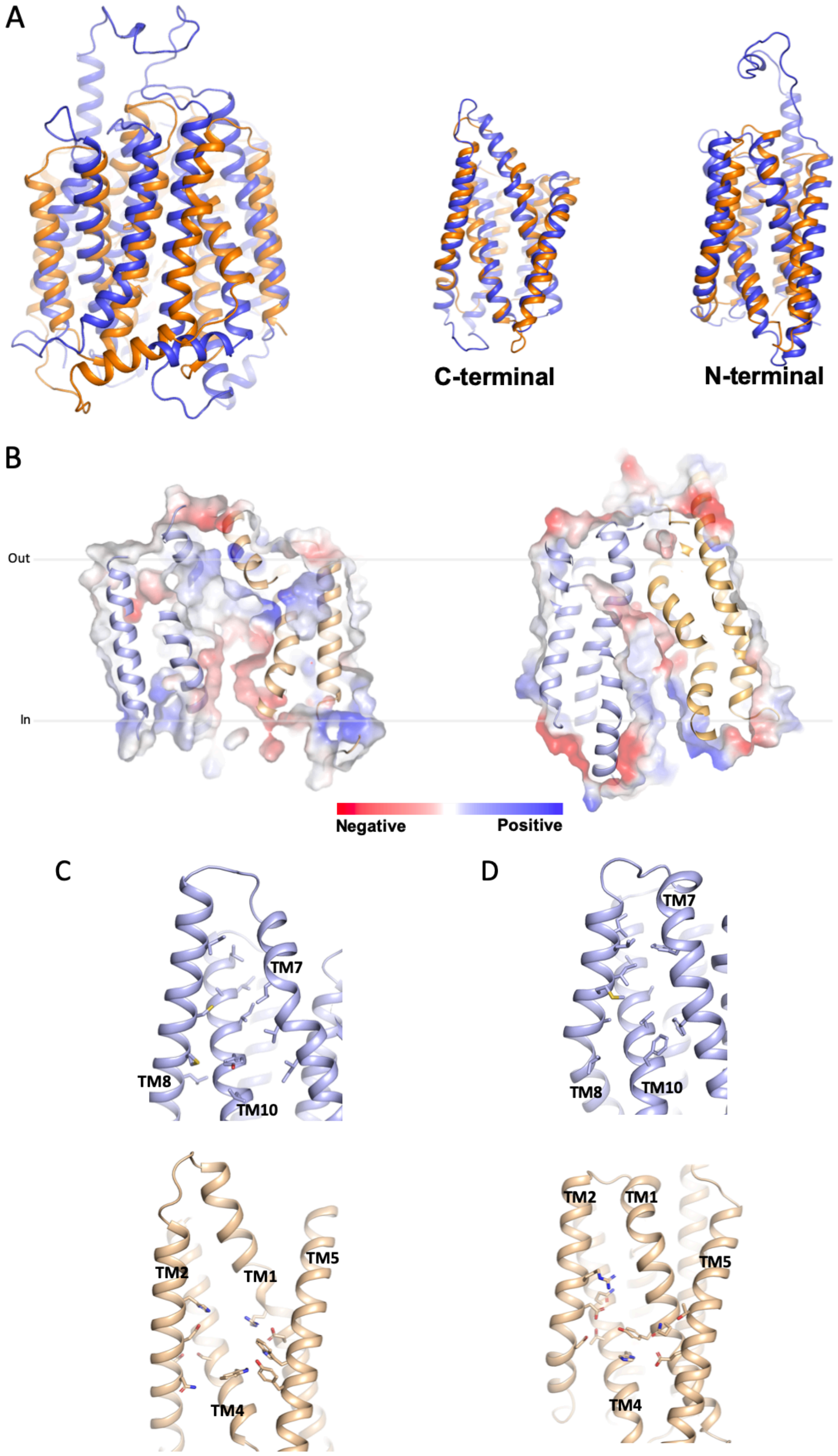

Supplementary figure 6. Comparison of LtaA and MFSD2A fold and amphipathic cavity. A. Superposition of LtaA (orange) and MFSD2A (blue) (r.m.s.d = 4.1Å), and individual N- and C-terminal domains (r.m.s.d= 2.6Å and 2.8Å, respectively). **B**. Vacuum electrostatic surface representation of inward-facing model of LtaA (*left*) and inward-facing MFSD2A (PDB ID 7MJS) (*right*). Residues participating in formation of N- and C-terminal hydrophilic and hydrophobic pockets in LtaA (**C**) and MFSD2A (**D**). N-terminal and C-terminal domains are shown in light orange and light blue, respectively.

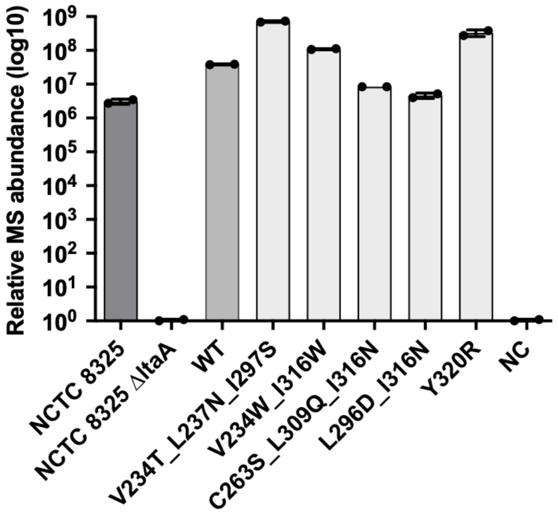

Supplementary figure 7. LC-MS analysis of relative abundance of LtaA-WT and variants in *S. aureus* membranes. The histogram shows relative abundances of LtaA WT and variants. Error bars show +/- s.d. of biological replicates (n=2).

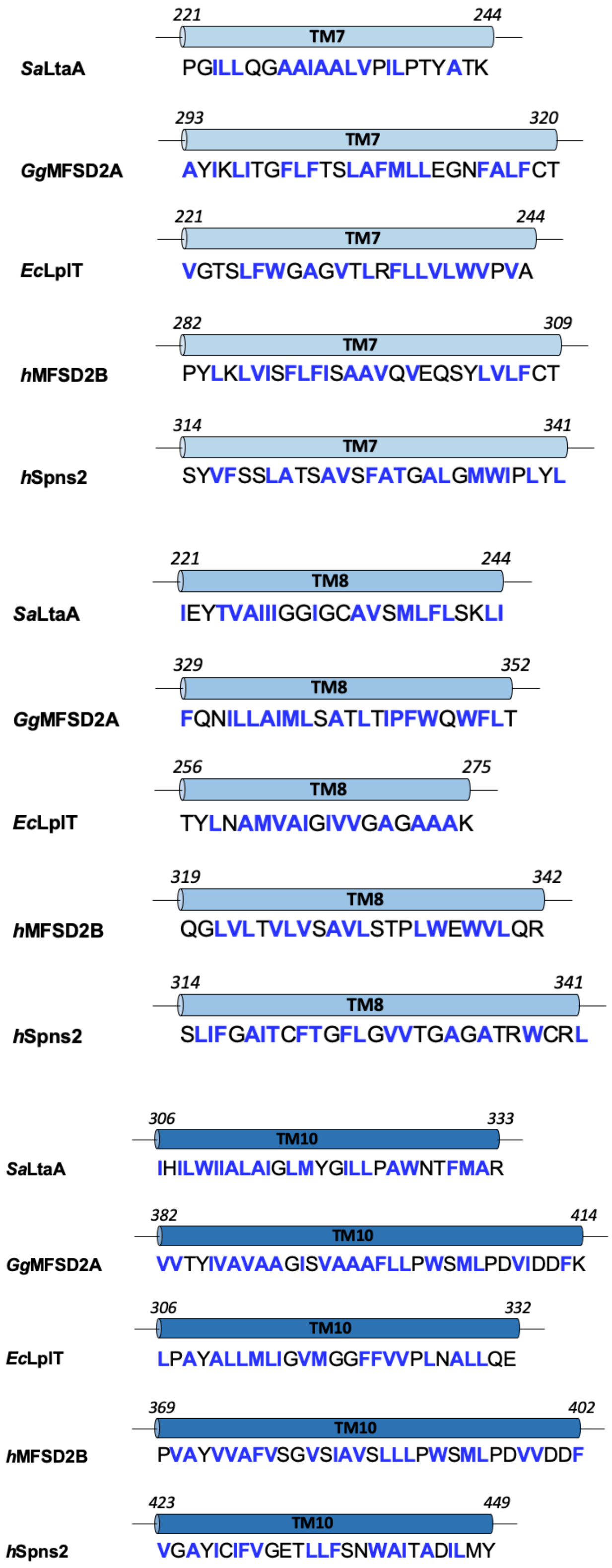

Supplementary figure 8. C-terminal TM helices participating in formation of hydrophobic pockets in MFS lipid transporters. Residues in TM7, 8, and 10 as seen in the structures of *S. aureus* LtaA (PDB ID 6S7V) and *Gallus gallus* MFSD2A (PDB ID 7MJS), and residues in predicted helical segments corresponding to the same TMs in *E. coli* LplT, human MFSD2B, and human Spns2 are shown. Hydrophobic residues are shown in blue.

Supplementary Table 1. Validation parameters of the top seven inward-facing models. *Initial*: homology models from Modeler: *SCWRL*; the refined model by SCWRL4; *mini*: energy minimization of the model in a bilayer using Gromacs.

Model	Clashscore			MolProbity score			Ramachandran favoured (%)			Ramachandran outliers (%)		
	Initial	SCWRL	mini	Initial	SCWRL	mini	Initial	SCWRL	mini	Initial	SCWRL	mini
1	144.2	125.8	11.5	3.46	2.89	2.03	95.5	95.2	93.4	0.8	1.0	1.0
2	155.0	125.3	11.5	3.49	2.89	2.15	95.2	95.2	92.9	0.8	0.8	1.6
3	145.5	125.1	10.3	3.45	2.91	2.3	94.9	94.9	92.2	1.1	1.1	1.6
4	137.9	117.8	12.0	3.25	2.85	2.05	95.5	95.5	92.9	0.5	0.5	0.8
5	144.9	119.1	10.6	3.34	2.89	1.98	94.9	95.0	93.4	0.8	0.8	1.3
6	134.7	125.7	8.12	3.35	2.89	2.17	95.2	95.2	93.6	1.1	1.1	1.1
7	131.8	125.5	9.81	3.31	2.86	2.18	95.7	95.7	92.3	1.1	1.1	1.1

Supplementary Table 2. Oligo's used in this study. Mutagenesis is underlined

Oligo name	5'-3' sequence
Sa-SalI-FW	GAGAGTCGACGGTCATTCATCACAACC
Sa-V234T-L237N-FW	GGTGCCGCAATTGCTGCCCTAACCCCTATAAATCCAACATATGCTACTAAGGTTAT
Sa-V234T-L237N-RV	ATAACCTTAGTAGCATATGTTGGATTTATAGGGGTTAGGGCAGCAATTGCGGCACC
Sa-V234W-FW	GTGCCGCAATTGCTGCCCTATGGCCTATATTACCAACATATGCTAC
Sa-V234W-RV	GTAGCATATGTTGGTAATATAGGCCATAGGGCAGCAATTGCGGCAC
Sa-C263S-FW	CATTATTGGTGGTATCGGCTCTGCAGTTTCGATGCTATTT
Sa-C263S-RV	AAATAGCATCGAAACTGCAGAGCCGATACCACCAATAATG
Sa-L296D-FW	TCTAAGCGGATTTATTTTATACATGATAGATATTTTTACTCTATCTATGATTGTTAATATTCACA
Sa-L296D-RV	TGTGAATATTAACAATCATAGATAGAGTAAAAATATCTATCATGTATAAAATAAATCCGCTTAGA
Sa-I297S-FW	GATTTATTTTATACATGATATTAAGTTTTACTCTATCTATGATTGTTAATATTC
Sa-I297S-RV	GAATATTAACAATCATAGATAGAGTAAAACTTAATATCATGTATAAAATAAATC
Sa-L309Q-I316N-FW	CTATGATTGTTAATATTCACATCCAATGGATTATCGCTTTAGCTAATGGTCTAATGTATGGCATCTT ATTAC
Sa-L309Q-I316N-RV	GTAATAAGATGCCATACATTAGACCATTAGCTAAAGCGATAATCCATTGGATGTGAATATTAACA ATCATAG
Sa-I316N-FW	CTTGTGGATTATCGCTTTAGCTAATGGTCTAATGTATGGCATCTTATTAC
Sa-I316N-RV	GTAATAAGATGCCATACATTAGACCATTAGCTAAAGCGATAATCCACAAG
Sa-I316W-FW	CTTGTGGATTATCGCTTTAGCTTGGGGTCTAATGTATGGCATCTTATTAC
Sa-I316W-RV	GTAATAAGATGCCATACATTAGACCCCAAGCTAAAGCGATAATCCACAAG
Sa-Y320R-FW	TTAGCTATCGGTCTAATGCGTGGCATCTTATTACCAGCATG
Sa-Y320R-RV	CATGCTGGTAATAAGATGCCACGCATTAGACCGATAGCTAA
LtaA-NotI-RV	GAGAGCGGCCGCAATAGTATTGTTAATCGTAGTATGTTTGAATTAATAAGA
F45C-FW	GAGCTACATTAACTGTCTGCCCACCG
F45C-RV	CGGTGGGCAGACAGTTAATGTAGCTC
A53C-FW	CCGTGACATCGATTTGTGTCGCTATTACATC
A53C-RV	GATGTAATAGCGACACAAATCGATGTCACGG
K80C-FW	CGTTATCGGCTTCTTATTATGTAAGTTTGGAACTAAGATC
K80C-RV	GATCTTAGTTCCAAACTTACATAATAAGAAGCCGATAACG
K141C-FW	GAGGATAAACGCGGCTGTCAAATGGGCTACGTG
K141C-RV	CACGTAGCCCATTTGACAGCCGCGTTTATCCTC
K166C-FW	CTTTATGAATTTGCTGATCTGTGTTCATCCTACTCGCTTCG
K166C-RV	CGAAGCGAGTAGGATGAACACAGATCAGCAAATTCATAAAG
I250C-FW	CAAAAGTCATTAATGTATCAACGTGTGAATATACCGTGGCTATTATC
I250C-RV	GATAATAGCCACGGTATATTCACACGTTGATACATTAATGACTTTTG
T253C-FW	GTATCAACGATTGAATATTGTGTGGCTATTATCATCGGCGG
T253C-RV	CCGCCGATGATAATAGCCACACAATATTCAATCGTTGATAC
C263S-FW	CGGCGGCATTGGATCGGCCGTTAGTATGTTG
C263S-RV	CAACATACTAACGGCCGATCCAATGCCGCCG
N276C-FW	CTTAGTAAGTTGATCGACTGCCGTTCGCGTAACTTTATGTAC
N276C-RV	GTACATAAAGTTACGCGAACGGCAGTCGATCAACTTACTAAG
E339C-FW	GCTTCATCAAGAGCGACTGTCAAGAGGAGACCTGG
E339C-RV	CCAGGTCTCCTCTTGACAGTCGCTCTTGATGAAGC
T366C-FW	GTTTGGCGGATTGATTTGCCAGTTCACCAAC
T366C-RV	GTTGGTGAACTGGCAAATCAATCCGCCAAAC
LtaA-V234T-L237N-FW	GATTGCGGCTTTGACTCCTATCAACCCTACGTATGCCACA
LtaA-V234T-L237N-RV	TGTGGCATACGTAGGGTTGATAGGAGTCAAAGCCGCAATC

LtaA-V234W-FW	GATTGCGGCTTTGTGGCCTATCCTGCCTAC
LtaA-V234W-RV	GTAGGCAGGATAGGCCACAAAGCCGCAATC
LtaA-C263S-FW	GCGGCATTGGATCGGCCGTTAGTATG
LtaA-C263S-RV	CATACTAACGGCCGATCCAATGCCGC
LtaA-L296D-FW	GGTTTTATTCTGTATATGATTGATATCTTCACGTTGTCTATG
LtaA-L296D-RV	CATAGACAACGTGAAGATATCAATCATATACAGAATAAAACC
LtaA-I297S-FW	TTATTCTGTATATGATTCTTAGTTTCACGTTGTCTATGATTGTC
LtaA-I297S-RV	GACAATCATAGACAACGTGAAACTAAGAATCATATACAGAATAA
LtaA-L309Q-FW	GTCAACATCCATATCCAATGGATTATTGCCTTGG
LtaA-L309Q-RV	CCAAGGCAATAATCCATTGGATATGGATGTTGAC
LtaA-I316N-FW	TGCCTTGGCGAACGGCCTGATG
LtaA-I316N-RV	CATCAGGCCGTTCGCCAAGGCA
LtaA-I316W-FW	ATTATTGCCTTGGCGTGGGGCCTGATGTAT
LtaA-I316W-RV	ATACATCAGGCCCCACGCCAAGGCAATAAT
LtaA-Y320R-FW	GATCGGCCTGATGCGTGGGATTCTGTTGCCA
LtaA-Y320R-RV	GATCGGCCTGATGCGTGGGATTCTGTTGCCA

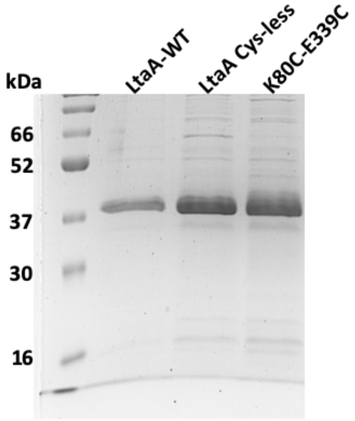

Suppl. Fig. 2B and 4B

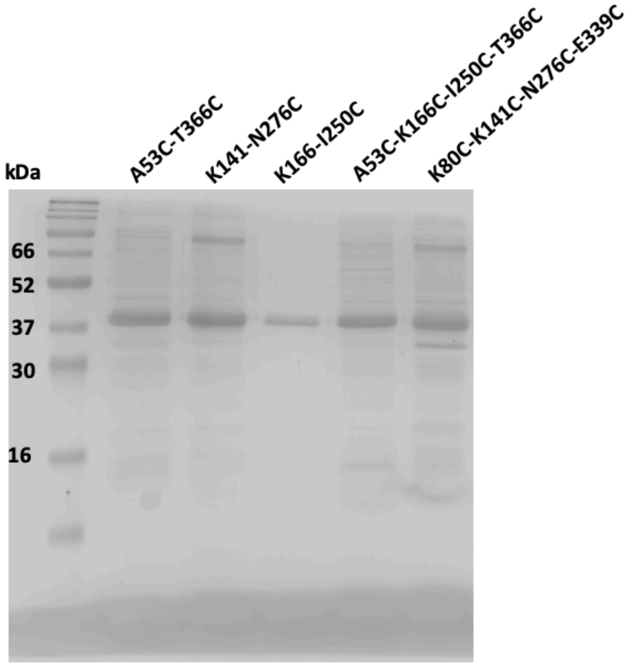

Suppl. Fig. 4B

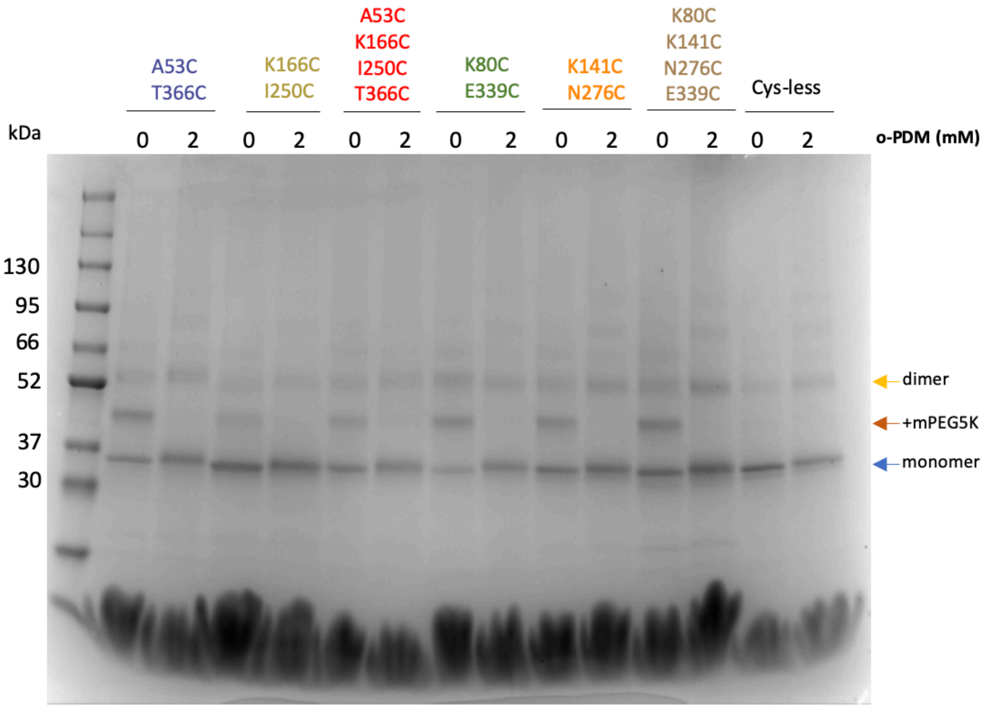

Fig. 2B and Suppl. Fig. 4C

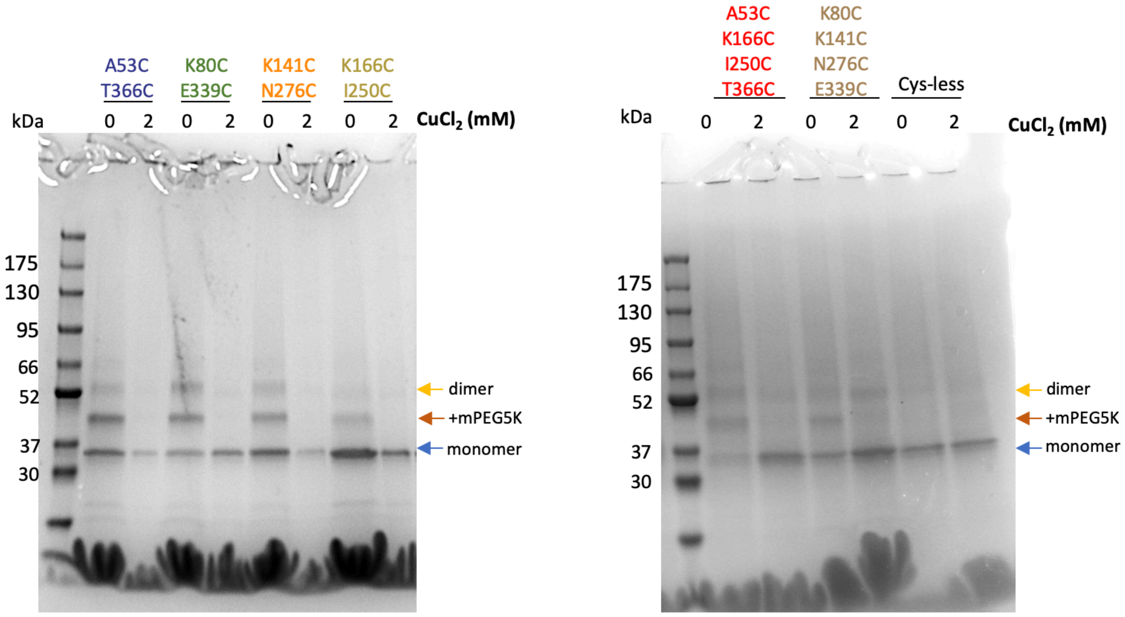

Fig. 4D and Suppl. Fig. 4D
