# Supplementary material for: Evidence for a trap-and-flip mechanism in a proton-dependent lipid transporter
Source: Nat Commun. 2022 Feb 23;13:1022. doi: 10.1038/s41467-022-28361-1 (PMC8866510; doi:10.1038/s41467-022-28361-1)
Supplement: Supplementary file 3 — Description of Additional Supplementary Files [file 41467_2022_28361_MOESM3_ESM.pdf]

Description of Additional Supplementary Files

File Name: Supplementary Movie 1
Description: Molecular dynamics simulations of outward-facing LtaA showing the intrusion of glycolipid and POPG molecules into the putative translocation pathway.

File Name: Supplementary Movie 2
Description: Molecular dynamics simulations of AF inward-facing LtaA showing the intrusion of glycolipid molecules into the putative translocation pathway.
